# Cyclic fatigue resistance of two NiTi rotary instruments after blue heat treatment

**DOI:** 10.4317/jced.63881

**Published:** 2026-04-25

**Authors:** Idalia Rodríguez-Delgado, Jennipher Monserrat Salazar-Vázquez, Jorge Jaime Flores-Treviño, Mayra Guadalupe Martínez-García, José M. Diabb-Zavala, Myriam Angélica De La Garza-Ramos

**Affiliations:** 1 Facultad de Odontología, Posgrado de Endodoncia, Universidad Autónoma de Nuevo León, Calle Dr. Eduardo Aguirre Pequeño y Sialo, Colonia Mitras Centro, Monterrey, 64460, Nuevo León México; 2 Facultad de Ingeniería Mecánica y Eléctrica, Universidad Autónoma de Nuevo León, Pedro de Alba s/n, Ciudad Universitaria, San Nicolás de los Garza 66455, Nuevo Leon, México

## Abstract

**Background:**

This study evaluated the cyclic fatigue resistance of two blue heat-treated rotary instruments. Thirty files (15 Eflex Blue and 15 BlueShaper) activated by an E-CONNECT motor at 350 RPM with a 2.5 N torque were evaluated.

**Material and Methods:**

A stainless-steel device with three different curvatures (25°, 45°, and 90°) was fixed to a SHIMADZU tensile testing machine to measure cyclic fatigue. The instruments were placed on the motor at a constant speed, then on the testing device; the time to each instrument's fracture was recorded. Tension, time, and instrument deformation were measured. Data were analyzed using the Shapiro-Wilk and Kruskal-Wallis tests at a 5% significance level.

**Results:**

Eflex Blue files demonstrated superior tensile strength and stability over time, particularly at a 25° curvature (p &lt; 0.05). Performance was comparable between the two files at 45° and 90° curvatures, with no significant differences in deformation percentage.

**Conclusions:**

Eflex Blue files demonstrated greater cyclic fatigue resistance than BlueShaper, especially at smaller curvatures (25°). Although both systems exhibited similar deformation rates, the fracture resistance of both files decreased as curvature increased, reaching a critical threshold at 90°.

## Introduction

Root canal preparation is an important phase of endodontic therapy aimed to remove pulp debris, microorganisms, and toxins through mechanical preparation and chemical disinfection (1 , 2). Mechanical preparation can be performed with manual or rotary instruments (3). Chemical disinfection and mechanical instrumentation of the root canal system are crucial for preventing or resolving apical periodontitis (4); therefore, the precision of root canal shaping is essential for endodontic treatment success (5). Since the introduction of Nickel-Titanium (NiTi) rotary instruments in 1992, the field has undergone significant evolution. Modern refinements, such as heat treatments and simplified file sequences, have enhanced file quality and mechanical strength (6). Modern files possess several favorable properties. NiTi alloys offer greater flexibility, torsional resistance, and enhanced corrosion stability. They also exhibit superelasticity, which allows these files to navigate complex root canal systems easily to achieve the required cleaning and shaping (2). The mechanical behavior of NiTi is determined by its design characteristics and NiTi superelasticity (or pseudo-elasticity), which lies in its metallurgical phase transition between the austenitic and martensitic crystal lattices. This stress- and temperature-dependent transition is defined by the austenitic finish temperature (7). This material has gradually replaced stainless steel due to its design, which includes tip size, taper, cross-section, helical angle, and coil spacing, which confer excellent flexibility, elasticity, and high cyclic fatigue resistance (8). NiTi files maintain the original canal shape, reducing procedural errors such as zipping and transportation (9). NiTi instrument fracture rates range from 1.3% to 10.0% (10); therefore, these instruments reduce procedural errors in narrow and curved canals (3). While conventional NiTi alloy is mainly in the austenite phase at room temperature (11). Thermal processing effectively modifies its transition temperatures. Alterations induced by heat treatment have been reported to significantly improve the flexibility and fatigue resistance of endodontic instruments (12). Several factors, including torsional fatigue, cyclic fatigue, or a combination of these, can influence NiTi endodontic rotary file fractures. A fractured endodontic rotary file can block a curved canal, negatively affecting treatment since disinfecting agents will no longer reach the infected root canal. Root canal systems that have not been properly disinfected are less likely to heal in periapical lesions (4). Other factors that can cause NiTi rotary file fractures include instrument cross-sectional design, taper diameter, apical flute length, pitch, and helix angle (13). Instrument dynamics, such as torque and canal geometry, and the manufacturing process-electropolishing, heat treatment, or ion implantation-can also influence fracture risk (4). Torsional forces and cyclic fatigue can cause unexpected NiTi instrument fracture in the root canal (14). Cyclic fatigue is the application of stress that produces deformation or fracture (4). Deformation or fracture occurs when root canal material is stressed by a small curvature radius, exceeding the instrument's flexibility, leading to cyclic fatigue. This fracture can even occur in morphologically straight and homogeneous canals; hence, the importance placed on the number of uses per file. Cyclic fatigue causes microcracks on the file surface. These begin as minimal defects on the outside of the instrument. Filing materials, design, geometric discontinuities, inclusions, porosities, and overheating during manufacturing can also lead to fatigue failures (1). This in vitro study evaluated the cyclic fatigue resistance of two different blue heat-treated rotary instruments with different curvatures.

## Materials and Methods

Thirty simulated canals were selected in a stainless steel model. Sample size was determined based on a quantitative assessment of central axis deviation in an infinite population using a 95% confidence interval, a standard deviation of 0.19, and a margin of error of 0.068. The result was 30 instruments divided into two groups: Group 1 consisted of 15 Eflex Blue files (Changzhou Sifary Medical Technology Co., Changzhou, China), and Group 2 of 15 BlueShaper files (Zarc4Endo, Gijón, Spain). The files were used to instrument three simulated canals with curvatures of 25º, 45º, and 90º, meeting the study's inclusion and exclusion criteria. The two clusters, Eflex Blue and BlueShaper, were activated with an E-CONNECT motor. Each system was operated at the manufacturer's recommended rpm and torque for rotary motion (Eflex Blue 350 rpm at 2.5 Ncm) and (BlueShaper 500 rpm at 4 Ncm). Cyclic fatigue was measured with a custom-designed stainless steel device with three distinct curvatures: 25°, 45°, and 90°. Each artificial canal measured 153 mm in length, 24 mm in width, and 89 mm in height, with a 2 mm curvature radius. The canals were engineered to a diameter of 1.5 mm and a depth of 1.5 mm. Measurements were performed using erosion, penetration, and high-precision milling to ensure better treatment quality and reliable experimental reproducibility. The file was fixed to the Shimadzu Model AG-X machine (1 kN-100 kN) and introduced into the E-CONNECT motor. The motor head was fixed to the Shimadzu machine to ensure a consistent force and pressure when introducing each file into the duct, so that all instruments entered at the same length. The fixed travel speed of the equipment was 50 mm/min. The seconds the instrument lasted until fracture were recorded, and the data were displayed on the graph at the exact moment of instrument separation. Once the instrument fractured, the graph was stopped, and all the data obtained during the examination was automatically recorded. The data was then organized into a Bluehill table. The acrylic cover on the stainless steel plate was unscrewed and reassembled to remove the separated fragment. This procedure was repeated for each evaluated instrument. The results were recorded in Excel spreadsheets for each analyzed group. The data were analyzed using the Shapiro-Wilk and Kruskal-Wallis tests at a 5% significance level.

## Results

The cyclic fatigue life of 30 rotary files (15 BlueShaper and 15 Eflex Blue) with blue heat treatment was evaluated in vitro with three curvature levels (25°, 45°, and 90°). Three variables, stress (N/mm²), time (min), and strain (%), were analyzed. The Shapiro-Wilk normality test was used for data analysis. Since the sample size was less than 30 and the p-values for the variables tension (p = 8.26 x 10-5) and time (p = 4.74 x 10-9) were less than 0.05, it was determined that they did not follow a normal distribution. Therefore, the nonparametric Kruskal-Wallis test was used for group comparison. This test showed statistically significant differences between groups for tension (p = 0.0027) and time (p = 0.0055), with no significant differences for deformation (p = 0.0987). The Eflex Blue file exhibited higher tensile strength than the BlueShaper file, especially at a 25° curvature, with an average of 30.35 ± 7.95 N/mm², compared with 11.70 ± 3.30 N/mm² for the BlueShaper. At 45° curvatures, Eflex Blue exhibited 11.20 ± 8.91 N/mm² and BlueShaper 6.28 ± 3.16 N/mm². At 90°, Eflex Blue decreased to 3.90 ± 1.03 N/mm², while BlueShaper reached 8.07 ± 1.48 N/mm² (Table 1).

Table 1

Statistically significant differences were observed between the groups (p &lt; 0.05). Regarding cyclic fatigue, Eflex Blue was more stable. At 25°, both files averaged 26.44 min, but at larger curvatures, BlueShaper showed greater variability (28.16 ± 3.44 min at 45°, and 26.13 ± 0.21 min at 90°), while Eflex Blue remained constant at 45° (26.42 ± 0.05 min) and showed a decrease at 90° (22.74 ± 4.22 min) (Table 2).

Table 2

The differences were significant (p &lt; 0.05) There were no significant differences in deformation between the two file brands. Both instruments showed similar percentages across the three curvature levels, ranging from approximately 22% to 23%, regardless of brand or curvature (Table 3).

Table 3

Therefore, it was concluded that the file type does not significantly affect deformation (%).

## Discussion

This study evaluated the cyclic fatigue resistance of two endodontic instrument systems using a specialized device that simulated clinical conditions with different root canal curvatures. The results indicate that the instrument systems differed significantly in their resistance to cyclic fatigue, with important implications for their clinical use. The methodology employed, which included using the E-CONNECT motor and instrument attachment to a Shimadzu machine, allowed for an accurate and controlled evaluation of cyclic fatigue resistance under standardized conditions. This finding is relevant because, as noted in the literature, static and dynamic testing offer different perspectives of endodontic instrument performance (15 , 16). Furthermore, the ProTaper Ultimate and BlueShaper systems exhibited good performance in terms of fractured fragment length, which is consistent with the expected properties of their respective Gold-Wire and Blue-Wire alloys. The relationship between fractured fragment length and cyclic fatigue remains an area of interest; however, this study did not find a statistically significant correlation. This result aligns with previous studies suggesting that fractured fragment length is not always a reliable predictor of cyclic fatigue resistance (17). Regarding the systems evaluated in this study, the Eflex Blue file showed higher tensile strength than BlueShaper, particularly at 25° curvature, suggesting better adaptability to less mechanically demanding trajectories. This finding can be attributed to the specific metallurgical properties of blue heat treatment, which affect the flexibility and shape memory of the material. These findings are consistent with studies highlighting how heat-treated alloys can significantly increase the cyclic fatigue resistance of rotary instruments (18). Regarding endurance time, Eflex Blue showed a more consistent and prolonged endurance than BlueShaper. However, performance decreased in both systems at 90°, consistent with established findings on the negative impact of severe curvature on instrument durability (19). Despite this, no significant differences in final deformation were observed between the two systems, indicating that plastic deformation was comparable across brands. These findings are consistent with the literature, which suggests that deformation is not always a direct predictor of structural failure (19). The BlueShaper system includes files with different tapers and heat-treated alloys (Blue), whose martensitic phase improves flexibility and resistance to cyclic fatigue (20). Although this property may influence its performance, the results obtained differ from expectations. This difference may be because the study was carried out on pieces extracted in vitro with variable, non-standardized curvatures and using irrigant (NaOCl). This study used a stainless steel model with three defined curvatures (25°, 45°, and 90°), with Eflex Blue showing better results in tension and stability. Because of the standardized nature of this study, several limitations must be acknowledged. First, the use of a static, stainless-steel artificial canal does not fully replicate the physiological conditions of human dentin or the complex, multi-planar geometries of natural root canals. Second, the tests were conducted under static rotation; however, in clinical scenarios, dynamic movement and the use of chemical irrigants may significantly alter the fatigue life of the instruments. Finally, environmental temperature, which influences the martensitic transformation of heat-treated NiTi alloys, was not controlled to mimic intracanal conditions.

## Conclusions

The Eflex Blue file showed higher tensile strength and more stable cyclic fatigue behavior than the BlueShaper at a 25° curvature. Both files showed similar levels of deformation, suggesting that heat treatment does not significantly influence this specific parameter. The resistance of rotary instruments decreases with increasing curvature, with 90° curvatures being the most critical.

## Figures and Tables

**Table 1:** Tensile strength (N/mm²) of BlueShaper and Eflex Blue files in different curvatures (25°, 45°, and 90°).

File type	Curvature (Degrees)	Tension (N/mm2)
BlueShaper	25	11.70 ± 3.30*
	45	6.28 ± 3.16
	90	8.07 ± 1.48
Eflex Blue	25	30.35 ± 7.95
	45	11.20 ± 8.91
	90	3.90 ± 1.03

Values are expressed as mean ± standard deviation. *Statistically significant differences were observed between the groups (p < 0.05).

**Table 2:** Average time (min) of BlueShaper and Eflex Blue files in different curvatures (25°, 45°, and 90°).

File type	Curvature (Degrees)	Time (min)
BlueShaper	25	26.44 ± 0.01*
	45	28.16 ± 3.44
	90	26.13 ± 0.21
Eflex Blue	25	26.44 ± 0.00
	45	26.42 ± 0.05
	90	22.74 ± 4.22

Values are expressed as mean ± standard deviation. *Statistically significant differences were observed between groups (p < 0.05).

**Table 3:** Deformation percentage (%) of BlueShaper and Eflex Blue files at different curvatures (25°, 45°, and 90°). Values are expressed as mean ± standard deviation.

Type of file	Curvature (ºC)	Deformation (%)
BlueShaper	25	22.00 ± 0.00*
	45	22.00 ± 0.00
	90	21.92 ± 0.11
Eflex Blue	25	22.00 ± 0.00
	45	22.00 ± 0.00
	90	21.23 ± 1.55

* Statistically significant differences were observed between groups (p < 0.05).

## Data Availability

The datasets used and/or analyzed during the current study are available from the corresponding author.

## References

[1] Al-Nasrawi SJH, Ayad Jaber Z, Talib Al-Quraine N, Imhemed Aljdaimi A, Jabbar Abdul-Zahra Al-Hmedat S, Zidan S, Haider J (2021). Impact of Peracetic Acid on the Dynamic Cyclic Fatigue of Heat-Treated Nickel-Titanium Rotary Endodontic Instrument. Int J Dent.

[2] Stošić N, Popović J, Anđ M, Mitić A, Nikolić M, Barac R, Kostić M (2023). Effects of Autoclave Sterilization on Cyclic Fatigue Resistance in 5 Types of Rotary Endodontic Instruments: An In Vitro Study. Med Sci Monit.

[3] Nouroloyouni A, Shahi S, Milani AS, Noorolouny S, Farhang R, Azar AY (2023). In Vitro Apical Extrusion of Debris and Instrumentation Time Following Root Canal Instrumentation with Reciproc and Reciproc Blue Instruments and a Novel Stainless Steel Rotary System (Gentlefile) Versus Manual Instrumentation. J Dent Res Dent Clin Dent Prospects.

[4] Faus-Matoses V, Faus-Llácer V, Ruiz-Sánchez C, Jaramillo-Vásconez S, Faus-Matoses I, Martín-Biedma B (2022). Effect of Rotational Speed on the Resistance of NiTi Alloy Endodontic Rotary Files to Cyclic Fatigue—an In Vitro Study. J Clin Med.

[5] Silva RV, Alcalde MP, Rodrigues CT, Silveira FF, Duarte MA, Nunes E (2021). Root Canal Shaping of Curved Canals by Reciproc Blue System and Pro Taper Gold: A Micro-Computed Tomographic Study. J Clin Exp Dent.

[6] Martins CM, Batista VEDS, Souza ACA, Andrada AC, Mori GG, Gomes Filho JE (2019). Reciprocating Kinematics Leads to Lower Incidences of Postoperative Pain Than Rotary Kinematics After Endodontic Treatment: A Systematic Review and Meta-Analysis of Randomized Controlled Trial. J Conserv Dent Endod.

[7] Peters OA, Arias A, Choi A (2020). Mechanical Properties of a Novel Nickel-Titanium Root Canal Instrument: Stationary and Dynamic Tests. J Endod.

[8] de Casco MG, Duarte MP, Boglio CFB, Castillo R (2022). Estudio Comparativo de Defectos Superficiales Producidos en Dos Limas Reciprocantes Sometidas a Diferentes Tratamientos Térmicos, Luego de su Uso en Raíces Mesiales de Primeros Molares Inferiores. Ex Vivo. Rev UniNorte Med.

[9] Shim KS, Oh S, Kum K, Kim YC, Jee KK, Chang SW (2017). Mechanical and Metallurgical Properties of Various Nickel-Titanium Rotary Instruments. Biomed Res Int.

[10] Del Pilar Matumay-Santos F (2020). Evaluación de la Resistencia a la Flexión de Limas Rotatorias Protaper Gold, 2Shape, V-Taper Fanta Gold. Rev Cient Odontol.

[11] Chan W, Gulati K, Peters O (2022). Advancing Nitinol: From Heat Treatment to Surface Functionalization for Nickel-Titanium (NiTi) Instruments in Endodontics. Bioact Mater.

[12] Elnaghy AM, Elsaka SE, Mandorah AO (2020). In Vitro Comparison of Cyclic Fatigue Resistance of TruNatomy in Single and Double Curvature Canals Compared with Different Nickel-Titanium Rotary Instruments. BMC Oral Health.

[13] Faus-Llácer V, Hamoud-Kharrat N, Marhuenda Ramos MT, Faus-Matoses I, Ruiz Sánchez C, Faus-Matoses V (2021). Influence of the Geometrical Cross-Section Design on The Dynamic Cyclic Fatigue Resistance of NiTi Endodontic Rotary Files—an In Vitro Study. J Clin Med.

[14] Hou XM, Yang YJ, Qian J (2021). Phase Transformation Behaviors and Mechanical Properties of NiTi Endodontic Files After Gold Heat Treatment and Blue Heat Treatment. J Oral Sci.

[15] Hülsmann M (2019). Research That Matters: Studies on Fatigue of Rotary and Reciprocating NiTi Root Canal Instruments. Int Endod J.

[16] Keleş A, Eymirli A, Uyanık O, Nagas E (2019). Influence of Static and Dynamic Cyclic Fatigue Tests on The Lifespan of Four Reciprocating Systems at Different Temperatures. Int Endod J.

[17] Karataslioğlu E, Aydın U, Yıldırım C (2018). Cyclic Fatigue Resistance of Novel Rotary Files Manufactured From Different Thermal Treated Nickel–Titanium Wires in Artificial Canals. Niger J Clin Pract.

[18] Plotino G, Grande NM, Cotti E, Testarelli L, Gambarini G (2014). Blue treatment enhances cyclic fatigue resistance of vortex nickel-titanium rotary files. J Endod.

[19] Rubini AG, Plotino G, Al-Sudani D, Grande NM, Putorti E, Sonnino G, Cotti E, Testarelli L, Gambarini G (2014). A New Device to Test Cutting Efficiency of Mechanical Endodontic Instruments. Med Sci Monit.

[20] Greco K, Iacono F, Montagna F, Esposito Corcione C, Paolone G, Gherlone E, Cantatore G (2024). Shaping ability of ProTaper Ultimate and BlueShaper in mandibular molars: a micro-CT evaluation. Front Mater.

